# Optimizing Bioaugmentation for Pharmaceutical Stabilization of Sewage Sludge: A Study on Short-Term Composting Under Real Conditions

**DOI:** 10.3390/jof11010067

**Published:** 2025-01-16

**Authors:** Gabriela Angeles-De Paz, Juan Cubero-Cardoso, Clementina Pozo, Concepción Calvo, Elisabet Aranda, Tatiana Robledo-Mahón

**Affiliations:** 1 Environmental Microbiology Group, Institute of Water Research, University of Granada, 18003 Granada, Spain; juan.cubero@ugr.es (J.C.-C.); clpozo@ugr.es (C.P.); ccalvo@ugr.es (C.C.); trobledo@ugr.es (T.R.-M.); 2 Department of Microbiology, University of Granada, 18003 Granada, Spain

**Keywords:** composting, sewage sludge, pharmaceuticals, eco-efficiency, biodegradation, bioaugmentation, toxicity

## Abstract

A significant concentration of pharmaceuticals has been detected within composted sewage sludge. Their uncomplete removal and lack of monitoring during composting neglects their potentially toxic effects when used as a soil organic amendment. Previously, we successfully implemented a bioaugmentation–composting system focused on toxicity and pharmaceuticals’ concentration reduction. This method, however, comprised a long inoculant-acclimatization period, making it an unprofitable technology. Hence, this work aimed to explore a shorter and yet effective composting process by simultaneously implementing the inoculation of a native microbial consortium and the fungus *Penicillium oxalicum* XD 3.1 in composting piles of sewage sludge and olive prunings. All the piles were subjected to frequent inoculation, windrow turning, and monitoring of the physicochemical and biological parameters. Additionally, both the bioaugmentation stability and pharmaceuticals degradation were evaluated through different analysis and removal rates calculations. One hundred days earlier than previous attempts, both bioaugmentation treatments achieved adequate composting conditions, maintained core native populations while improving the degrading microbial diversity, and achieved around 70–72% of pharmaceutical remotion. Nevertheless, only *Penicillium* inoculation produced favorable toxicity results ideal for organic amendments (acute microtoxicity and phytotoxicity). Thus, a shorter but equally stable and effective degrading bioaugmentation–composting with *P. oxalicum* was achieved here.

## 1. Introduction

Pharmaceuticals entering the environment is the unavoidable consequence of their consumption, manufacturing, excretion, and improper disposal, all of which contribute to their presence in sewage systems without exemptions [1]. Their environmental presence is mainly determined by population growth and demographics. Indeed, global medicine usage has been recklessly raised by 14% over the past five years (2019–2024), and a further 12% increase is expected through 2028, bringing annual use to 3.8 trillion defined daily doses [2]. Moreover, other aspects, such as health systems, manufacturing sectors, connectivity to sewage treatment systems, and the establishment of effective regulatory frameworks, could significantly increase exposure risk [3].

Many attempts at chemical, physical, biological, and even social interventions have been proposed to mitigate or decrease these pharmaceuticals throughout their life cycle and mobilization, from their production to their stabilization when accumulated in sewage sludge [1,4,5]. The latter is a current subject matter of the circular economy and SDGs 2023 Agenda, which has encouraged governments, institutions, and researchers from all over the world to implement strategies focused on the evaluation, management, and valorization of sewage sludge towards its recirculation for agricultural purposes [6,7]. Nutrient recycling, secondary resource providing, and environmental and health protection have become the priority aims of stabilization procedures, including alkaline and biological techniques. Among all of these, composting is a biological decomposition procedure which offers a 3500 GWh energy recovery, a reliable method to eliminate pathogenic bacteria, and a good alternative for soil fertilizers production [8]. Organic matter degradation and mature compost sanitization is ensured due to the temperature fluctuation (which sets the three main stages of composting as mesophilic, thermophilic, and maturing) and the microbial succession responsible for the temperatures (mesophilic bacteria and fungi, thermophilic bacteria dominated by the order Bacillota, and mesophilic actinomycetes and cellulolytic fungi) [9]. Nevertheless, composting achieves a partial or uncomplete removing of pharmaceuticals, even with a long-term composting time [10].

The introduction of microorganisms through bioaugmentation has been shown to enhance the traditional composting of sewage sludge by increasing enzyme production, improving micropollutant degradation, and breaking down complex molecules, resulting in higher-quality compost with reduced phytotoxicity [11]. Compared with physical and chemical treatment methods, a bioaugmentation approach for sewage sludge composting has produced ideal results, as its application in in situ works has improved the microbial counts in complex matrices; introduced and identified specialized microorganisms for specific degradation purposes; and offered better stability, sustainability, low energy consumption, low production of toxic by-products, and high efficiency of xenobiotics removal [12]. Although bioaugmentation can improve and optimize composting, many uncertainties and difficulties concerning its effectivity in real applications have been reported. Overall, the incompatibility of bioaugmentation attempts with scale-up procedures are owing to (i) the lack of activity, persistence, and compatibility of the inoculants to a new environment and its native microorganisms; (ii) the limited knowledge about the load of the initial inoculum and application frequency; (iii) the unmonitored toxic substances causing biotoxicity within the location of the study; and (iv) its long-term operation and, thus, maintenance and development costs [13,14].

Based on the drawbacks mentioned above, two different inoculants were previously tested to determine their ability to improve sewage sludge composting (focused on pharmaceuticals removal) using a natural microbial consortium (NMC) and the fungus *P. oxalicum* XD 3.1 [11]. In contrast to the NMC, *P. oxalicum* XD 3.1 is an exogenous inoculant which was isolated from a pond contaminated with aliphatic and aromatic hydrocarbons [15]. It has proven pharmaceutical-degrading capabilities, versatility, great adaptation to reactor-scale experiments, and microbial social skills [16,17].

The bioaugmentation followed a two-tandem methodology: the first cycle was set for the inoculant’s adaptation and the second for the degradation of organic matter and contaminants. In general, the inoculant enabled the degradation of persistent compounds, such as carbamazepine, cotinine, and methadone, whilst it removed a wider variety of the total pharmaceuticals compared to conventional composting (21% more) [11]. However, 220 days of operation were needed to achieve such results. Longer composting cycles require more land and specialized labor, and they also influence gaseous emissions. Therefore, the present study aimed to reduce the general process by applying a simultaneous inoculation implementation (SII), which consisted of the integration of the two cycles previously established while ensuring a complete biological stabilization, pharmaceuticals removal, toxicity reduction, and minimizing the general cost of the procedure.

## 2. Materials and Methods

### 2.1. Obtaining Inoculants

Prior to the composting assay, two distinct microbial inoculants were obtained and labeled according to the inoculant type: (i) natural microbial consortia (NMC) and (ii) exogenous fungus strain (*Penicillium oxalicum* XD 3.1). Details of their acquisition and identification have been published by Aranda et al., 2017; Ledezma-Villanueva et al., 2022; and Angeles-de Paz et al., 2024 [15,18,19]. The NMC was obtained through a top–down enrichment methodology with raw sewage sludge [18] in modified Kirk medium (glucose 5 g L^−1^, yeast extract 1 g L^−1^, peptone 1 g L^−1^, ammonium tartrate 2 g L^−1^, KH_2_PO_4_ 0.2 g L^−1^, MgSO_4_·7H_2_O 0.5 g L^−1^, KCl 0.5 g L^−1^, mineral solution 1 mL L^−1^, and vitamins supplement 1 mL L^−1^) supplemented with 100 µM diclofenac, ketoprofen, and 17-β-estradiol over a 9-week incubation period (28 °C, 120 rpm). The resulting consortium was massively produced in 5 L Erlenmeyer flasks whose pellets were concentrated and resuspended in 30 L of tap water. *P. oxalicum* spores were collected from Malta Extract Agar (MEA, VWR chemicals, Radnor, PA, USA) cultures after 5 days of incubation at 28 °C. The final concentration was adjusted to 6.25 × 10^9^ spores kg^−1^ of sludge using a Neubauer chamber and resuspended in 30 L of piped water.

### 2.2. Composting Design and Sample Collection

The composting material used to build the piles included digested sewage sludge (dSS) provided by the EMASAGRA (Granada, Spain) wastewater treatment plant in Granada, and olive leaf prunings as the bulking agent (B). Three equal piles (labeled NMC-P, P-P, and C-P, according to the treatment) were then placed at the facilities of the Environmental Complex EIDER recycling Eco-industry (coordinates: 37.32583820223778, −3.08280105397221) in Guadix, Granada, Spain, with the ensuing dimensions: 5 m (length) × 3 m (width) × 2 m (height). Their assembly comprised 8 tons of dSS and the respective volumetric amount of B (1:3 *v*/*v* dSS: B). Each pile was subjected to bioaugmentation (frequent inoculation at 0, 7, 14, 30, 60, and 120 days during composting) throughout the experiment until sieving, and named after the inoculants (NMC pile, *Penicillium* Pile). A control pile was also included and conventionally watered (control pile). The experimental period lasted 160 days, including composting, sieving, and maturation (from December 2020 to May 2021). The three compost piles underwent regular mechanical turning and continuous monitoring over the operation time.

Timing of sample collection for physicochemical and biological monitoring occurred at the piles building (T0); 7, 14, 30, and 60 days after; sieving of the piles (T120); and piles removal (T160), from which a 3 kg composite sample was obtained. Each composite resulted from the combining of individual sub-samples taken from dig cross sections throughout each windrow at 0, 25, 50, 75, and 100 cm depths. A portion of the sample was immediately used for physicochemical parameters (Section 2.3) and total colony-forming units (CFUs) (Section 2.5.1). The rest of the sample was stored at −20 °C and −80 °C for subsequent analysis of enzymatic activity, microbial diversity, phytotoxicity, and pollutant content (pharmaceutical active compounds and heavy metals) (Section 2.4, Section 2.5.2, Section 2.5.3 and Section 2.6.2, respectively).

### 2.3. Physicochemical Parameters Monitoring

Standardized methodologies published as Normalized Working Procedures described in ’The official methodology Ministry of Agriculture vol III’ and ’Official bulletin from the state agency’, Spain, as well as ’The Official Journal of the European Union’ were used to monitor the general parameters of each pile [7]. These parameters included daily temperature measurement (using a portable sensor at three core locations), pH, moisture, total and volatile solids, and macronutrient content. Additionally, pathogen presence (*Escherichia coli* and *Salmonella* sp.) was assessed according to the most probable number technique (MPN) as specified by the ISO 16649-1:2018 [20] and ISO 6579-1:2017 [21].

### 2.4. Pollutants Determination

Pharmaceutical active compounds (PhACs) were extracted and measured from 200 g of the composite samples according to their chemical nature as previously described by Montemurro et al., 2021 [22], at the Institute for Water Research Foundation of Catalonia (IDEA-ICRA). Briefly, PhACs were analyzed chromatographically by a Waters ACQUITY UPLC (Waters, Milford, MA, USA) system coupled to a Q-Exactive mass spectrometer (Thermo-Fisher Scientific GmbH, Dreieich, Germany) and equipped with a heated electrospray ionization (HESI) chamber. A C18 column (Waters ^®^ ACQUITY UPLC ^®^ HSS T3) of 100 mm × 2.1 mm i.d., 1.8 μm particle size was injected with 10 μL of each sample at a column temperature of 40 °C. Conditions of the isocratic and gradient elution are detailed in [22]. Data quantification was performed using Thermo TraceFinder 5.1 software (Thermo-Fisher Scientific GmbH, Dreieich, Germany) with the internal standard method.

For heavy metal (HM) determination, 100 g samples were analyzed before and after composting. Total mercury content was assessed using EPA 7473 with a Direct Mercury Analyzer DMA-80 Milestone ^®^ (Brondby, Denmark). Hexavalent chromium (Cr VI) was measured spectrophotometrically at 540 nm using a UV-Vis Spectrophotometer (Agilent, Santa Clara, CA, USA). The remaining metals (Cu, Zn, Pb, Cd, and Ni) were processed after digestion with HCl + HNO_3_ following the Spanish version of European CEN methods using flame atomic absorption spectrometry (AENOR, UNE-EN 13650 [23], PerkinElmer, Waltham, MA, USA).

### 2.5. Biological Parameters

#### 2.5.1. Total Count of Culturable Microorganisms

On arrival at the laboratory, the total CFU count of culturable composite fungi and bacteria were performed using a dilution plate-count technique. First, 1 g of composite was suspended in 9 mL of 0.45% and 0.9% NaCl for fungi and bacteria, respectively. Afterward, aliquots of 100 µL from dilution series 10^−3^ and 10^−5^ were inoculated for plate counting in Malt Extract Agar (MEA; VWR, Radnor, PA, USA) medium with 50 µg L^−1^ of tetracycline and incubated at 28 °C for 7 days for fungi CFU determination. For the bacterial CFU counting, 100 µL from dilution series 10^−6^ and 10^−8^ were inoculated in Tryptic Soy Agar (TSA; Oxoid Ltd.™, Basingstoke, UK) with 50 µg L^−1^ of cyclosporine and incubated at 30 °C for 24 h. In all cases, CFUs were then counted and expressed per gram of composite sample (CFU g^−1^).

#### 2.5.2. Enzymatic Activity

The activity of five different enzymes (dehydrogenase, protease, β-glucosidase, phosphatase, and arylsulfatase) were determined by triplicate with colorimetric methods based on the protocols presented in Table 1, using a Unicam 5625 UV/VI spectrophotometer (Unicam, Budapest, Hungary). A reference curve was used for quantification at the end of each enzymatic activity determination. Results are presented as µg^−1^ h^−1^ per unit of dry compost weight. Enzymatic activity is presented as quantity of enzyme required to convert 1 µg of substrate per time (µg^−1^ h^−1^) per gram of dry compost.

#### 2.5.3. Microbial Succession, α and β Diversity

To maximize the yield of purified and high-quality DNA from the composite samples, a treatment prior to DNA isolation was performed in 50 mL DNAse and pyrogen-free centrifuge tubes ^®^ (Thermo Fisher Scientific, Waltham, MA, USA). Briefly, it consisted of serial soaking with 1X phosphate-buffered saline (PBS) pH = 7,4 and concentrating through sonication and centrifugation (800× *g* for 10 min) [18]. An ultimate pellet was obtained after one final centrifugation at 4696× *g* for 10 min.

The DNA extraction was executed as indicated by the manufacturer instructions in the FastDNA™ Spin kit for Soil (Palex Medical, SA, Sant Cugat del Valles, Barcelona, Spain). To confirm the DNA concentration and purity, we used a NanoDrop ND-1000 spectrophotometer (Thermo Fisher Scientific, Waltham, MA, USA). The extracted DNA pool was sequenced using Illumina MiSeq technology at StarSEQ GmbH (Mainz, Germany) employing the sequencing primers ITS2_fITS7 Fw (5′ GTGARTCATCGAATCTTTG 3′), ITS4 Rev (5′ TCCTCCGCTTATTGATATGC 3′), 16S ProV3V4 Fw (5′ CCTACGGG-NBGCASCAG 3′), and 16S ProV3V4 Rev (5′ GACTACNVGGGTATCTAATCC 3′) for the library construction of fungi and bacterial communities.

Bacterial taxonomy was assigned based on Silva v138.1 data bank [29] after ASVs quality filtering and trimming using FIGARO to maximize reads retention [30]. Due to the highly variable length of ITS sequences, filtering and trimming were modified according to DADA2 ITS pipeline workflow v1.8 [31]. The forward and reverse fungal reads were first cleaned using the adapter alignment algorithm Cutadapt [32]. During quality filtering, the argument minLen was enforced to supply truncation to a fixed length, which was inappropriate for ITS processing. The reads were finally merged to be gathered into amplicon sequence variants (ASVs) and subsequently screened for chimeras by a de novo detection. The UNITE ITS v8.3 database was used for molecular identification of fungi taxa [33]. Raw data were stored in NCBI’s Sequence Read Archive with the following Bio Project accession number: PRJNA780876.

The diversity analysis consisted of calculating the feature richness as a direct ASV count, Shannon diversity/entropy, and Simpson dominance index by using the alpha module from SKBIO package. The vegan package in R was employed to visualize and assess community compositional differences (beta diversity) through NMDS. The ordination patterns were acceptable, as the stress values of the two-dimensional NMDS analysis were below 0.2000. NMDS stress values were reported after 100 tries and the best solution was repeated 40 times.

### 2.6. Toxicity Bioassays

#### 2.6.1. Microtox^®^ Bioassay

Microtox^®^ bioassay (Microtox^®^ Model 500 Toxicity Analyzer, Madrid, Spain) was used to determine the acute toxicity of the samples as previously described [11]. Prior to the analysis, the samples were treated by employing a solid–liquid extraction with 2% NaCl and 5 µg L-1 NaHCO_3_ solution at pH = 7 ± 0.2. The microtoxicity test was conducted with a Microtox^®^ bioassay (Microtox^®^ Model 500 Toxicity Analyzer, Madrid, Spain) and the results were expressed as EC_50_, defined as the concentration causing a 50% reduction in luminescence of marine bacterium *Aliivibrio fischeri* after 5 min and 15 min of exposure.

#### 2.6.2. Phytotoxicity Bioassays

The phytotoxicity test consisted of the germination index (% GI) of the watercress *Lepidium sativum* [34]. First, non-viable seeds were discarded after 1 h of hydration with tap water whilst composite samples were diluted with distilled water (1:1, 1:2, 1:5, and 1:10 *w*/*v*) and shaken for 1 h at 250 rpm. Glass Petri dishes of 9 cm diameter, previously cleaned with ethanol and sterilized by dry-heat sterilization, were used to carry out the germination experiment. Each Petri dish was filled with 2 mL of each dilution, covered with filter paper (Watman no 2) until its complete absorbance, and finally 20 seeds were distributed over it. The dishes were dark-incubated for 48 h at 28 °C. Each sample unit was tested in triplicate. A control set with tap water was also included. Finally, the % GI (Equation (3)) was calculated considering the relative seed germination (% RSG, Equation (1)) and the relative radicle growth (% RRG, Equation (2)) using the following equations:

% RSG = (G/G_0_) * 100 (1)

% RRG = (L/L_0_) * 100 (2)

% GI = (% RSG/% RRG) * 100

where G is the number of germinated seeds with the sludge dilution, G_0_ is the number of germinated seeds in the control set, L is the length of the radicle in the germinated seeds within the sludge dilution, and L_0_ is the length of the radicle in the seeds germinated in the control set.

### 2.7. Statistical Analysis

All experiments were performed in triplicate with complete randomization. Data are presented as means ± standard deviations. One-way repeated-measures ANOVA was employed for emerging pollutant concentrations. Heavy metal content and phytotoxicity bioassay data, enzymatic activity, and CFU differences were analyzed using two-way repeated-measures ANOVA, while Microtox^®^ (Toxicity Analyzer, Madrid, Spain) results were assessed with a two-way ANOVA. Pairwise comparisons were conducted with Tukey’s multiple range test, assuming normality and homoscedasticity of raw data. Significance was set at *p* < 0.05 using SigmaPlot v15.0 (Grafiti LLC., Palo Alto, CA, USA).

## 3. Results and Discussion

### 3.1. Physicochemical Parameters Succession During Composting Experiments

**Figure 1:**
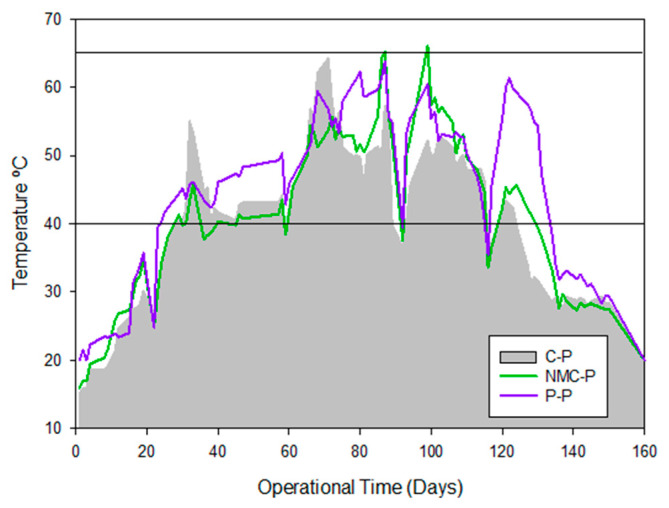
Temperature monitoring during operation time of composting in piles C-P (control pile), NMC-P (natural microbial consortium pile), and P-P (*Penicillium* pile).

Based on the temperature, three main phases (mesophilic, thermophilic, and maturation) were identified during the composting process, whose duration and intensity varied according to the bioaugmentation treatment employed (Figure 1). Both the NMC-P and P-P underwent thermophilic conditions for longer periods (Table 2) and reached higher temperatures than the conventional composting shown in the C-P (Figure 1). Greater temperatures could lead to improved decomposition outcomes by (i) changing the parameters linked to moisture evaporation and organic matter degradation, and (ii) enhancing the sanitization of the compost by eliminating the most common pathogens in the sewage sludge [35]. Longer thermophilic periods, on the other hand, support thermophilic microorganisms’ survival and their degradation mechanisms through enzymatic activity with more efficacy [36].

All the mature compost presented with physicochemical features within the preferred intervals that ensure good performance when applied in soils (Table 2), which, according to different authors, include values for pH between 5.5 and 7.2, electrical conductivity <4 dS m^−1^, moisture content ± 30%, TOC between 8 and 35%, C/N ratio < 20, and total N between 1 and 3% [37,38]. Organic matter losses at the end of composting were 10% in the C-P, 14% in the NMC-P, and 31% in the P-P. These OM losses were calculated based on the increase in ash content, or mineral matter, using the equation provided by Paredes (2020) [39]. The nutrient-rich character of sewage sludge has been widely reported; however, stabilization treatments have often registered important losses of essential elements (N, P, K, Mg, and Ca) due to their natural tendency to leach given their high solubility [40], which was nevertheless not the case here (Table 2). Indeed, a significant increment in NPK and Mg was observed in the mature compost of both bioaugmented treatments. The P-P, particularly, showed a greater increase in Ca (296%) compared to 209% in the NMC-P and 214% in the C-P. Previous studies have proved the positive correlation between Ca ions and the salt and heavy metal stress relieved during seed germination and growth in different crops fertilized with urban SS rich in Ca [41].

Enteric pathogens (*Escherichia coli* and *Salmonella* sp.) sanitization must be ensured by the composting processes, and it was accomplished by all the treatments (Table 2), indicating their good sanitation and effectiveness due to the temperatures reached in all the treatments [42].

### 3.2. Removal of Micropollutants During the Composting Process

**Figure 2:**
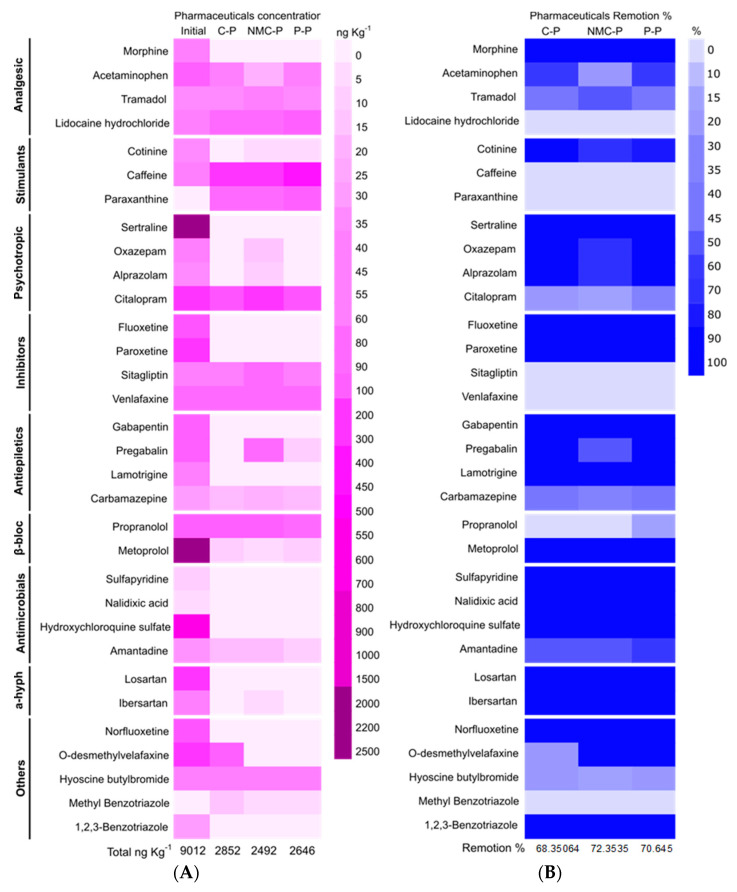
Heatmap of pharmaceutical active compounds concentration in ng g^−1^ (**A**) and % of remotion (**B**) in composite samples at the beginning of the process (initial) and at the end of the process. C-P: control pile; NMC-P: natural microbial consortia; P-P: *Penicillium* pile.

Of the 72 pharmaceuticals targeted, only those detected in the starting material (initial) were classified and listed according to their pharmacological effect in Figure 2. All the treatments achieved a great percentage of remotion (80–100%) of most of the psychotropics, antimicrobials, and antihypertensives (a-hyph), including the most abundant compounds in the initial sample (sertraline and metoprolol). Although these PhACs are easy to degrade by traditional composting methods, a significant amount of recalcitrant compounds was still detected in the C-P mature compost, including amantadine and acetaminophen. The bioaugmentation treatments (NMC and *Penicillium*), on the other hand, successfully achieved a partial removal of these compounds.

Amantadine is an anti-influenza viral drug that, contrary to other antivirals, is highly resistant to biotransformation and to photodegradation during composting [43]. Its adsorption throughout the fungal hyphae, membranes, and vacuoles might be inferred since it is a common protection response of several fungi against different xenobiotics. Antiviral mechanisms are, nevertheless, not well defined and are still largely unknown [44]. Contrary to this, acetaminophen degradation has been highly reported via four main microbial metabolization pathways (via p-acetanisidine, 4-aminophenol, 3-hydroxyacetaminophen, and catechol) during composting associated with different species of bacteria and fungi, including *Penicillium* [44].

Unlike “two-steps composting”, where carbamazepine, methadone, and gabapentin are removed [11], the bioaugmentation proposed here showed different degradation targets. The common chemical and physical characteristics among the PhACs, besides all the environmental factors and conditions modified by the composting itself, hindered specificity about the degradation mechanisms caused by the NMC and *Penicillium* [45]. However, the results concerning *ο*-desvenlafaxine degradation remain consistent between both techniques under a *Penicillium* treatment (two-steps and simultaneous bioaugmentation–composting), whose persistence during photodegradation and biotransformation makes it hard to eliminate in sludge.

In general, the classes of pharmaceuticals affected by bioaugmentation differed in both techniques regardless of the inoculant. However, similar removal percentages of the total PhACs concentration were achieved under both bioaugmentation–composting technologies (73.69% NMC-P and 76.56% P-P, and 72.35% NMC-P and 70.65% P-P under two-steps and simultaneous composting, respectively) [11]. Considering the significant reduction in operation time and costs that simultaneous composting requires and the small difference between the percentage of removal under the best treatment (6% in the P-P), the presented optimization can be considered as a more cost-effective process for pharmaceuticals degradation through bioaugmentation with the NMC and *P. oxalicum*.

In Table 3, the accumulated concentration of the different HMs in each treatment before and after composting is displayed. According to the current law BOE-A-2022-23052 [46], all the mature compost obtained from the three different treatments tested here (C-P, NMC-P, and P-P) correspond to a soil fertilizer in class C limiting its use in degraded soils (caused by prior contamination activities like mining, manufacturing, and the textile industry). However, the P-P mature compost presents with a lower accumulative concentration of HMs and a removal rate above 40%. Although they are non-biodegradable micropollutants and their removal from sewage sludge is often questioned, bioaugmentation results have contributed to the attribution of bioleaching as a proven microbial pathway able to solubilize metal oxides and sulfides [47]. The microorganisms involved in the bioleaching process are mostly acidophilic and chemolithoautotrophic iron- and/or sulfur-oxidizing bacteria [48], but the most recent research has found great leaching efficiency and recovery with the fungi from genera *Aspergillus* and *Penicillium* via biotransformation and chemical bounding with extracellular polymeric substances [49]. Bioleaching using *P. oxalicum* has not been deeply explored yet. Nevertheless, a previous report of reducing Cu by 50% and Zn by 38% after 220 days of composting was published [11], which, compared to the individual rates achieved under the present conditions, were significantly higher. This, however, owes its efficiency to the lower initial concentration of HMs (778.9 mg Kg^−1^) and the length of operation time [25]. According to Dusengemungu et al. [49], the adaptability of filamentous fungi to metal-polluted areas represents an advantage for bioremediation purposes; however, their capacity to tolerate multi-metal exposure is constricted by high concentrations and multifactorial stresses under real and industrial conditions. When using bioaugmentation techniques, the inoculant needs to adapt to new environments, multiple contaminants, native populations, and so on, which might vary from place to place [50]. Thus, large-scale bioleaching procedures are still far from being understood, optimized, and reproduced.

In any case, both HMs and pharmaceuticals have a strong relationship and shared fate when they take part in co-contaminated environmental matrices. Elevated Cu concentrations delay the dissipation of Sulfa-class drugs, resulting in their longer half-lives [51]. This phenomenon highlights the importance and urgency to propose, optimize, and prove new strategies for targeting HMs and pharmaceuticals simultaneously during composting that might also be liable to be cost-effective and routine procedures.

### 3.3. Biological Parameters: Fungal and Bacterial CFUs, Enzymatic Activities, and Microbial Communities’ Succession

**Figure 3:**
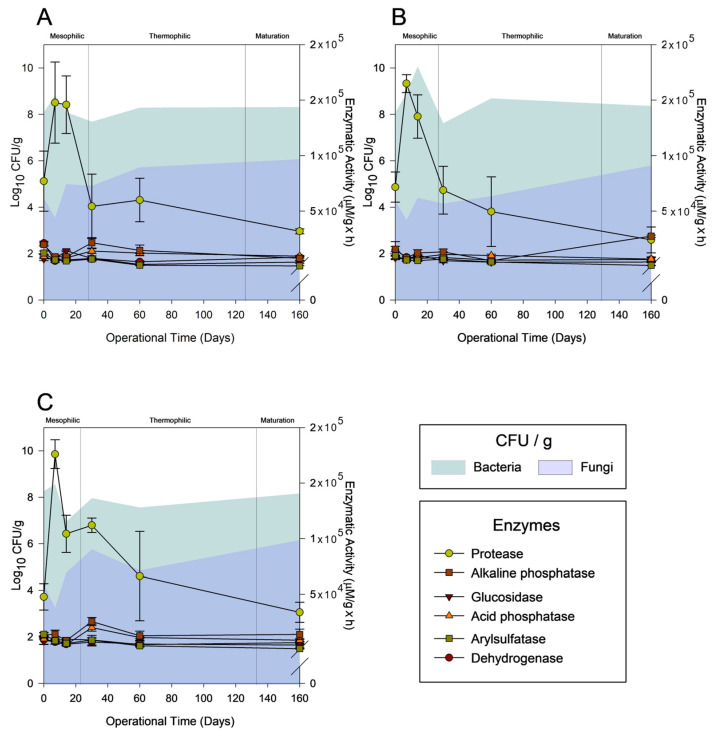
Biological parameters throughout the composting process in the different treatments: (**A**) control, (**B**) NMC, and (**C**) *P. oxalicum* XD 3.1, considering both the culturable fungi and bacteria and enzymatic activity (protease, alkaline phosphatase, glucosidase, acid phosphatase, arylsulfatase, and dehydrogenase). Error bars indicate the standard deviation of three replicates (*n* = 3).

The progression of the bacteria and fungi CFUs and their enzymatic activity are represented in Figure 3. In the control pile (Figure 3A), the highest hydrolytic activity and number of bacteria and fungi are achieved under mesophilic conditions but decrease with rising temperatures, and are finally reconstituted and maintained after 60 days of composting. The biological parameters succession under the NMC-P treatment does not show any differences in comparison to the control (Figure 3B). The treatment with *P. oxalicum* XD 3.1 presents a similar trend but is observed earlier in the process, after just 30 days of composting, suggesting a faster organic matter decomposition (Figure 3C). By this time, the P-P has reached temperatures between 45 and 50 °C, and yet the culturable microorganisms prevail at the same levels as the those in the control. Some authors have reported that the number of organisms under these temperature conditions are about three times lower than the number of those seen under mesophilic temperatures, mainly caused by the exhaustion of oxygen and the inactivation of metabolism functioning, especially as the denaturalization of enzymes and nutrients reduces availability [52]. However, as confirmed by plenty of studies, various species of *Penicillium* have been demonstrated to produce hydrolytic enzymes in a wide range of temperatures whose metabolic systems are closely related to the nature of organic compounds, their stability, and properties at the given temperature and pH [53,54,55]. Particularly, the strain *P. oxalicum* XD 3.1 can increase protease and other hydrolytic enzymes in a composting system, as well as stimulate other microbial species with degrading abilities [11,55].

Protease and alkaline and acid phosphatase activities were significantly higher under the P-P compared to the other treatments during the thermophilic stage (Figure 3). Inoculation with *Penicillium* seemed to affect these enzymes through different paths, considering their characteristics during the composting process, including the following: (i) offsetting the negative feedback inhibition by NH_3_ production and (ii) improving the diversity and abundance of thermophilic proteolytic enzyme-secreting microorganisms [56,57]. Favorable conditions could then occur during these stages that affect the microbial communities and their further effect on pharmaceuticals degradation. Indirectly, inoculation with *P. oxalicum* could affect enzyme activities by modifying the HMs and pharmaceutical concentration throughout composting. For instance, a low concentration of HMs promotes enzyme activity during the early periods of composting. However, high concentrations will disrupt the structure of enzymes.

**Figure 4:**
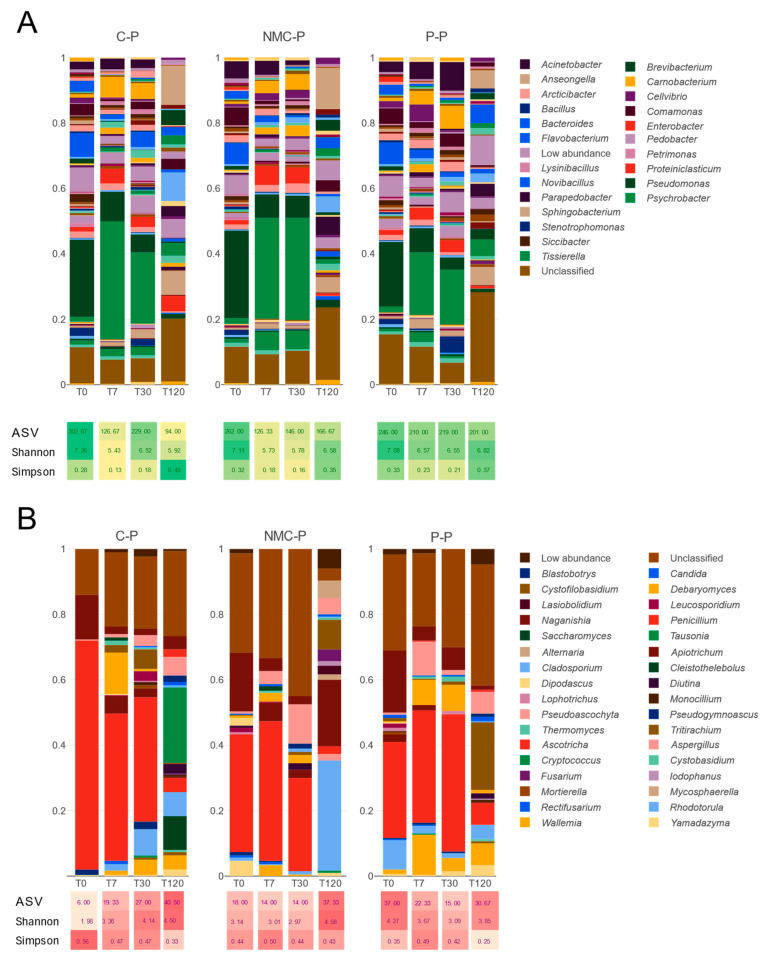
Relative abundance of bacteria (**A**) and fungi (**B**) throughout the composting process in the three different piles. C-P: control pile; NMC-P: natural microbial consortium; and P-P: *Penicillium* pile. The bacterial genera with relative abundance < 0.03% were excluded from the legend. Richness (ASVs) and alpha diversity indexes (Shannon and Simpson) are shown below the sampling time.

Luo et al., 2023 [58] summarized the succession of microbial communities during the different stages of composting as follows: first, mesophilic bacteria and fungi are subject to cellulose decomposing, and finally to lignin-decomposing microorganisms. This trend has been observed and reported throughout the years by several composting studies using different input components for the composting mixture, with the specific composition strongly influenced by the latter [59]. Similarly, in Figure 4, the microbial composition in the C-P is mainly shaped by the temperature and the physicochemical parameters fluctuations recorded during the composting. At temperatures below 40 °C (Tt0–T7), the composting process is dominated by mesophilic bacteria, primarily *Pseudomonas* (23%) and *Flavobacterium* (7%) (Figure 4A), as well as fungi, including *Penicillium* (70%) and *Apiotrichum* (13%) (Figure 4B). These genera, recognized for their roles as phosphate-solubilizing bacteria (PSB) or phosphate-solubilizing fungi (PSF) [59,60], dominate over others but decrease in abundance at higher temperatures. Their wide number of enzyme activities could contribute to the initial decomposition of organic matter in the early stages of composting [36]. The high microbial activity and massive production of metabolites and the degradation of compounds from the original substrates create a new physicochemical environment and thermophilic phase (at T30). The thermophilic and thermotolerant microbial bacteria have a maximum relative abundance at 46–50 °C (*Psychrobacter,* 21%; *Pseudomonas,* 5.4%; *Carnobacterium,* 6%; and *Proteiniclasticum,* 4%) (Figure 4A), while the fungal species *Penicillium* (38%), *Rhodotorula* (8%), and *Cystofilobasidium* (6%) tolerate high temperatures (Figure 4B). These microorganisms presumably can metabolize complex organic matter components and have shown a strong relation with xenobiotics degradation, i.e., halogenated compounds, microplastics, and pharmaceuticals via hydrolytic enzymes [61]. At the maturation stage, the mesophilic populations re-emerge, including bacterial genera such as *Anseongella* (11%), *Novosphingobium* (8%), *Sphingobacterium* (7%), and *Pedobacter* (5%), as well as fungal species like *Saccharomyces* (10%), *Rhodotorula* (7%), and *Aspergillus* (5%). These microorganisms are well known for their roles in breaking down cellulose, carboxymethylcellulose, hemicellulose, xylan, and arabinoxylan, contributing to the stability and maturity of compost [62,63].

The bioaugmentation with both inoculants maintained the core microbiota and succession observed in the C-P (Figure 4A,B, NMC-P and P-P), but the distribution of the major taxa and their dominance varied according to the conditions caused by the inoculants and the inoculants *per se*. For instance, during the NMC treatment, the low-abundant taxa (in the C-P) *Tisierella* (5%) and *Acinetobacter* (4%) maintained their abundance occupancy from T7 to T30. For the specific xenobiotic degradation events that occur during the composting process, they might play an important role as a part of the “functional core-microbiota”, since it has been reported that both bacteria can hydrolyze complex insoluble substrates into smaller molecules and belong to the key phyla (Bacillota and Pseudomonadota) associated with the degradation of organic compounds [64]. Particularly, the genus *Tisierella* seems to have strong evidence of its resistance to and degradation of sulfadiazine and other pharmaceuticals [65]. The biggest rearrangement of dominancy observed among the fungi was that of the genus *Aspergillus,* which ended covering around 42% of the abundance at T30. Its early reconstitution during composting, regardless of the temperature, and its association with high cellulolytic enzymes activity might be a clear sign of an earlier maturation of the compost [66].

Similarly, the *Penicillium* treatment in the P-P maintained the abundance of the different taxa from the functional core microbiome (previously reported as pharmaceutical degradation microorganisms, Aguilar-Romero et al., 2024 [67]), such as some thermophiles, including *Psychrobacter* (19%), *Carnobacterium* (6%), *Proteiniclasticum* (3.6%), and *Acinetobacter* (8%), and others with the poorest abundancy (<3%), including *Comamonas* (4%), *Cellivibrio* (5%), and *Stenotrophomonas* (4.9%). Compared to the C-P and NMC-P, the dominant taxa of fungi were more evenly distributed during the bioaugmentation–composting (*Penicillium,* 42%; *Debaryomyces,* 8%; *Wallemia,* 12%; and *Aspergillus,* 10%), despite the challenging environment between T7 and T30, where their enzymatic machinery is normally reduced [36]. More degrading microorganisms lead to more chances for effective performance and more enzymatic activity, microbial survival, and ions mobility, and thereby the better quality of the matured compost [68].

The diversity and richness of both the bacteria and fungi experienced changes throughout the composting, being especially less diverse during the unfavorable conditions until maturation, where diversity was restored (Figure 4A,B, C-P). Since the NMC-P was subjected to higher temperatures due to the bioaugmentation, the effect on diversity was more evident, expressed as lower ASVs and Shannon indexes as compared to the C-P (Figure 4A,B NMC-P). Despite the physicochemical similarities between both bioaugmented treatments observed in Table 1 and contrary to what is expected from conditions over 65 °C [68], the diversity of the P-P microbial populations remained stable during the whole experiment, with higher values of ASVs and the Shannon and Simpson indexes (Figure 4A,B, P-P). The inoculation with *P. oxalicum* was thus beneficial by providing protection from adverse conditions that could include but are not limited to salinity, drought, temperature, and heavy metal stresses, while improving the media for other fungi development according to previous research [69].

One of the biggest drawbacks found in bioaugmentation processes is the general reduction in microbial diversity, which has demonstrated that the inoculum used becomes the dominant species in the contaminated area [14]. Nevertheless, neither the core microbial composition of the NMC [19] nor *P. oxalicum* accomplished a particular increment of their own population within the compost. Moreover, the results lead us to conclude that they work synergistically with each other and the native microbiota, which helps to increase microbial abundance and diversity, thus contributing to accelerating the humification process and improving the quality of the final product [70].

**Figure 5:**
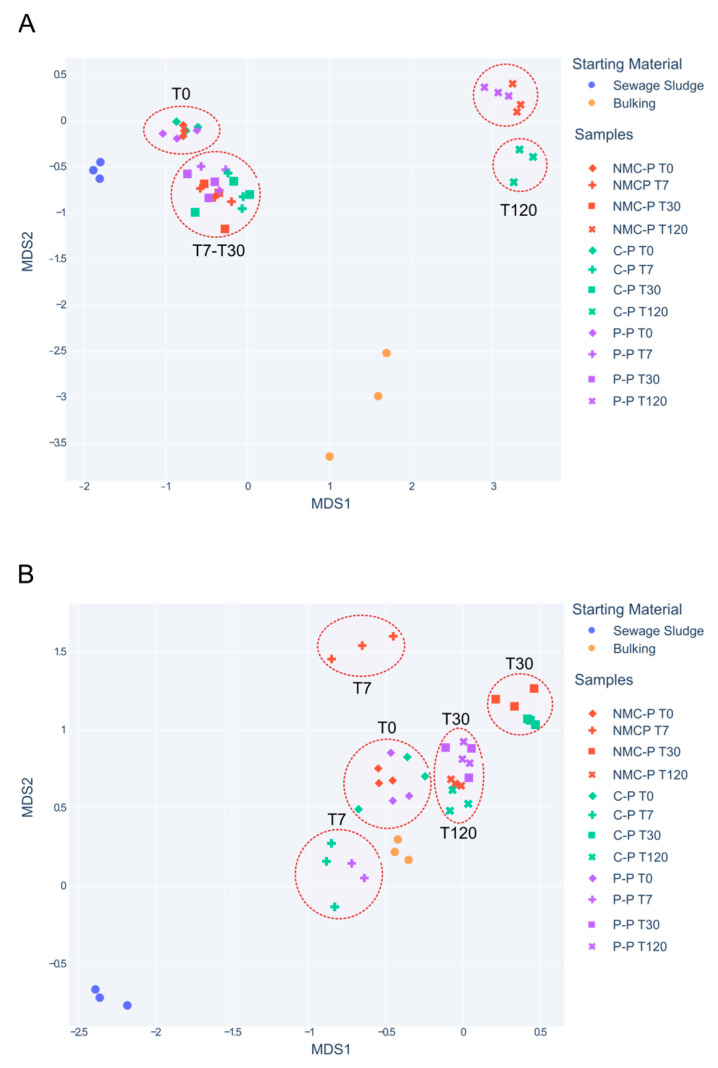
Nonmetric multidimensional scaling (NMDS) for bacterial (**A**) and fungal (**B**) community composition between three different treatments. C-P: control; NMC-P: enriched culture; and P-P: *Penicillium* pile. Sampling times are marked with different symbols. Each sampling time is highlighted with dotted circles.

In Figure 5, the microbial composition dissimilitude is studied through nonmetric multidimensional scaling (NMDS) to analyze the differences between the communities due to the inoculation frequency and quantity (an insufficient inoculum amount may result in its depletion by the native communities, while a larger load may disrupt the microbial distribution within the remediated location) [71]. On one hand, the addition of the NMC and *P. oxalicum* selectively modifies the abundance and function of some microorganisms, as discussed previously, but it upholds the general bacterial composition throughout the experiment (Figure 5A). The fungal communities, on the other hand, exhibit important compositional dissimilarities between the treatments at T7 and T30, but ended closely related to the C-P at T120 (Figure 5B). These results follow the distribution previously described for a shorter inoculation procedure, where some inoculants were introduced into sewage sludge composting piles over 60 days [19]. Thus, we conclude that both the NMC and *P. oxalicum* XD 3.1 triggered the emergence of specific functional fungal species, which acted on the entire system to optimize the removal of other pollutants and the operating parameters of the system, and maintained the core microbiome within the sewage sludge, regardless of the inoculation frequency.

### 3.4. Toxicity Bioassays

**Figure 6:**
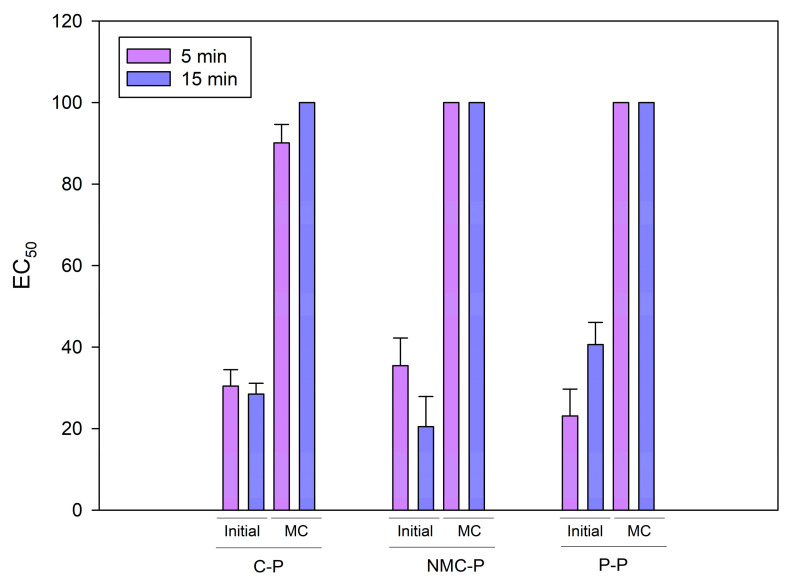
Acute toxicity (as EC_50_) of composite samples from all treatments before (initial) and after (MC: mature compost) the bioaugmentation–composting process. C-P: control pile; NMC-P: natural microbial consortia; P-P: *Penicillium* pile. Error bars indicate standard error from mean (*n* = 3).

The EC_50_ values of the initial and the mature compost extracts from each treatment are shown in Figure 6. The initial extracts show high toxicity while the mature compost extracts (from all treatments) did not affect the bioluminescence activity even after 15 min of exposition. The results of the C-P and P-P are consistent with those obtained from a long-term bioaugmentation–composting [11]. On the other hand, the NMC inoculation methodology presented here produces better results with no effect on *Aliivibrio* bioluminescence. The pharmaceuticals and HMs that underwent biotransformation and long-range mobilization by the NMC could cause the formation of unforeseen and uncharacterized chemicals which, under a longer operation time, could lead to an accumulative effect on the mature compost, resulting in higher toxicity [72]. In addition, within the solubilizing fraction of the composted sewage sludge, other concerning compounds could be present, including biological pollutants (mycotoxins, antibiotics, virucides, fungicides, anti-nematodes, and proteins) and other contaminants non-explored in the employed methodology (microplastics) [73].

**Figure 7:**
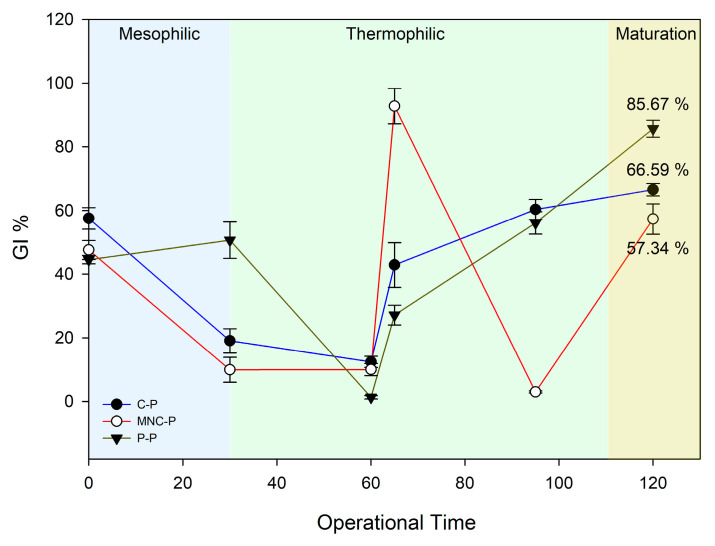
Phytotoxicity of composites samples from all treatments throughout bioaugmentation–composting process. C-P: control pile; NMC-P: natural microbial consortia; P-P: *Penicillium* pile. Final germination indexes (GI %) are written above bars. Error bar indicates standard error from mean (*n* = 3).

The seed germination index (%GI) was used as an indicator of compost maturation (Figure 7). While the GI encompasses a broad range of causes, the impact of micropollutants on plant health remains a common theme [74]. Many authors have examined the relationship between the contaminants present in composted sludge and their phytotoxicity mechanisms. For instance, heavy metals inhibit plant hormone activity (responsible for germination) through binding competition, overstimulating free radicals (ROS) production resulting in enzyme malfunction, and interrupting the double-stranded DNA structure [75]. Pharmaceuticals of different classes delay seed germination, possibly due to the interruption of superoxide dismutase gene expression and affecting the ROS concentration, which promotes the breaking of dormancy and mobilization reserve [76]. Regarding the pharmaceuticals mainly removed with *Penicillium* bioaugmentation (amantadine and o-desvenlafaxine) and their metabolite derivates, phytotoxicological information is still scarce. However, their presence seems to indicate a deep relationship with ROS generation and further effects on other eukaryotes [75].

## 4. Conclusions

In this work, we have successfully implemented a bioaugmentation–composting system based on simultaneous inoculation implementation (SII) with a natural microbial consortium and the exogenous fungus *P. oxalicum*. For both inoculants, all the parameters reflected the stability of the compost piles assisted by bioaugmentation, where adequate physicochemical and biological characteristics were satisfactorily reached to classify the mature compost as a Class C organic fertilizer. Among the inoculants, SII with *P. oxalicum* remained the best for bioaugmentation, as its seed acted as a reliable and stable inoculant. In this study, it has been demonstrated that *P. oxalicum* does not need an adaptative stage to show positive effects on sewage sludge composting and it is able to operate under different stress conditions (a higher initial concentration of micropollutants, competition with native microorganisms, and a longer and higher thermophilic stage). In addition, the *Penicillium* inoculant produced favorable germination results ideal for organic amendments, making SII with *P. oxalicum* a more cost-effective bioaugmentation. Consequently, its use translates to a reduction in operational costs compared to other bioaugmented approaches (about 100 days less), while keeping the parameters stable and improving the degradation performance in a similar time compared to conventional composting.

## Figures and Tables

**Table 1:** Details of the enzymatic activity procedures and their references.

Enzyme	Substrate	Reaction	λ nm	Reference
Dehydrogenase EC: 1.1.1.2	2,3,5-triphenil tetrazolium	Reduction in the substrate to triphenyl formazan (TPF)	485	Casida et al. [24]
Protease EC: 3.4.2.21–24	Sodium casein solution	Hydrolysis of the substrate to casein formation	700	Ladd and Butler [25]
β-glucosidase EC: 3.2.1.21	ρ-nitrophenyl-β-D-glucopyranoside	Quantification of ρ-nitrophenol	400	Eivazi and Tabatabai [26]
Acid and Alkaline Phosphatase EC: 3.1.3.2 and 3.1.3.1	ρ-nitrophenyl-phosphate	Quantification of ρ-nitrophenol at pH = 6.5 and 11, respectively	400	Tabatabai and Bremner [27]
Arylsulfatase EC: 3.1.6.1	ρ-nitrophenyl sulphate	Quantification of ρ-nitrophenol	400	Tabatabai and Bremner [28]

**Table 2:** Physicochemical parameters of the initial material and all treatments at the beginning and the end of the composting.

				Treatments					
				C-P		NMC-P		P-P	
Temperature	Days in mesophilic phase (10–40 °C)			28		27		23	
	Days in thermophilic phase (40–65 °C)			98		102		110	
	Days in maturation phase (40–18 °C)			34		31		27	
		**Initial Material**		**C-P**		**NMC-P**		**P-P**	
		**dSS**	**B**	**T0**	**T160**	**T0**	**T160**	**T0**	**T160**
General Characteristics	pH	7.9	5.4	7.9	7.2	8.3	7.1	7.8	7.4
	Conductivity (dS/m)	0.7	1.6	0.7	1.8	0.6	1.7	0.6	1.4
	Humidity (% p/p)	81.9	3.9	72.4	27.2	74.3	25.7	76.3	31.1
	Dry Matter	18.0	95.6	27.6	72.8	25.7	74.3	23.7	68.9
	Mineral Matter (%)	5.1	15.1	13.3	36.9	13.3	41.3	10.5	36.9
Macronutrients	Total %N (p/p)	1.1	0.9	1.2	2.3	1.0	2.1	1.1	1.8
	%P (p/p)	1.1	0.2	1.1	2.9	1.2	3.3	1.3	2.8
	%K (p/p)	0.04	0.5	0.1	0.6	0.1	0.7	0.1	0.7
	%Ca (p/p)	0.8	6.4	3.2	6.8	3.5	7.3	2.3	6.9
	%Mg (p/p)	0.4	0.3	0.5	1.5	0.5	1.5	0.5	1.5
	Total	3.5	8.2	5.9	14.2	6.3	14.8	5.3	13.6
Organic Compounds	Total Organic Matter (%)	12.2	80.4	14.3	35.9	12.4	33.0	13.2	32.0
	Dry Organic Matter (%)	70.5	84.2	51.8	49.3	48.2	44.4	55.7	46.4
	Total Organic Carbon (%)	7.1	46.6	8.3	20.8	7.2	19.2	7.6	18.6
	Dry Organic Carbon (%)	40.9	48.8	30.1	28.6	28.0	25.8	32.3	26.9
	C/N	6.4	51.2	7.2	9.0	7.0	9.1	7.2	10.4
CFU g^−1^	*Escherichia coli*	4200	90	>15,000	<10	>15,000	<10	>15,000	<10
	*Salmonella* sp.	n.d	n.d	n.d	n.d	n.d	n.d	n.d	n.d

n.d: non-detected.

**Table 3:** Heavy metal concentration (mg Kg^−1^) contained in the mature compost under the three composting treatments.

Heavy Metal	Initial	C-P	NMC-P	P-P
Cu	808.3 ± 98.7	386.3 ± 32.5	325.0 ± 55.2	333.3 ± 43.5
Zn	420.8 ± 54,8	329.3 ± 2.1	411.3 ± 2.8	350.0 ± 18.4
Cr	30.2 ± 8.8	27.0 ± 2.1	25.0 ± 1.4	26.7 ± 4.2
Ni	40 ± 12	33.7 ± 0.7	32.7 ± 0.7	32.0 ± 1.1
Pb	55 ± 10	33.7 ± 2.1	36 ± 4.2	32.7 ± 1.4
Hg	0.6 ± 0.01	0.3 ± 0.002	0.4 ± 0.001	0.3 ± 0.001
Accumulative Concentration	1356.23	810.28	830.36	774.94
% removal		40.26	38.78	42.87

## Data Availability

The original contributions presented in the study are included in the article, further inquiries can be directed to the corresponding authors.
